# Explanations and information-giving: clinician strategies used in talking to parents of preterm infants

**DOI:** 10.1186/s12887-016-0561-6

**Published:** 2016-02-11

**Authors:** M. E Redshaw, M. E Harvey

**Affiliations:** National Perinatal Epidemiology Unit, University of Oxford, Oxford, UK; Department of Perinatal Imaging and Health, Division of Imaging and Biomedical Engineering, King’s College, 4th Floor North Wing, St Thomas’ Hospital, SE1 7EH London, UK; Faculty of Health, Education and Life Sciences, Birmingham City University, Birmingham, UK

**Keywords:** Communication, Clinician strategies, Parents, Neonatal brain imaging, Information-giving, Neurological prognosis

## Abstract

**Background:**

The study is part of a larger research programme on neonatal brain imaging in the trial element of which parents were randomised to receive prognostic information based upon either magnetic resonance imaging (MRI) or ultrasound findings (ePrime study). The aim of this study was to investigate the strategies used by clinicians in communicating with parents following imaging at term age of the brain of preterm infants born before 33 weeks gestation, focusing on explanations and information-giving about prognosis

**Method:**

Audio recordings of discussions between parents and clinicians were made following MRI and ultrasound assessment. Parents were given the scan result and the baby’s predicted prognosis. A framework was developed based on preliminary analysis of the recordings and findings of other studies of information-giving in healthcare. Communication of scan results by the clinicians was further explored in qualitative analysis with 36 recordings using NVivo 10 and the specifically developed framework. Emerging themes and associated sub-themes were identified.

**Results:**

The ways in which clinicians gave information and helped parents to understand were identified. Within the over-arching theme of clinician strategies a wide range of approaches were used to facilitate parental understanding. These included orienting, checking on previously acquired information, using analogies, explaining terminology, pacing the information, confirming understanding, inviting clarification, answering parents’ questions and recapping at intervals. Ultimately four key themes were identified: ‘Framing the information-giving’, ‘What we are looking at’, ‘Presenting the numbers and explaining the risk’ and ‘Appreciating the position of parents’.

**Conclusions:**

The interviews represent a multifaceted situation in which there is a tension between the need to explain and inform and the inherent complexity of neurological development, potential problems following preterm birth and the technology used to investigate and monitor these.

**Electronic supplementary material:**

The online version of this article (doi:10.1186/s12887-016-0561-6) contains supplementary material, which is available to authorized users.

## Background

Relatively little research has been carried out on giving parents technical clinical information in relation to diagnosis and prognosis. Qualitative research has focused more generally on clinician-parent communication [1, 2] and a systematic review of interventions used in communicating with and supporting parents of preterm infants focused on a range of information-giving methods including ward rounds, notes based discussions, websites and written information [3]. Audio recordings of discussions between parents and clinicians in the neonatal unit have previously been described [4–6]. However, the main purpose and effectiveness of such recordings was to facilitate parental recall rather than investigating the nature of the communication process itself.

Communication about neonatal brain imaging was explored in a small-scale study of parents’ experiences in the neonatal unit [7]. Concerns about long-term developmental outcome were evident and the effect of having a preterm infant had a negative impact on parents’ ability to retain information at this time. A recent account of one couple’s experience of information-giving after an MRI at term of their preterm baby [8] and the clinicians’ responses to their account [9] raised the issue of MRI not necessarily being beneficial for parents in this context.

In a small scale study of seven families of children with dysmorphic features communication of information was explored [10]. Content and discourse analysis, following verbatim transcription of recorded consultations, indicated that the discussion elements focusing on challenging issues such as the child’s appearance and longer term outcome were more negative. On these occasions lack of fluency, more repetition, clinician difficulty finding suitable terminology and an imbalance in clinician and parent participation in the discussion were noted. The contributions made by parents were generally more open, direct and goal focused, leading to the authors to suggest that parents may prefer a more frank approach [10]. Another study used audio-recording of clinician-parent communication to analyse discussions about their child’s possible participation in a clinical trial, finding that clinical staff rarely asked open questions and parents said little [11]. However, no published studies were identified which have used audio recording to specifically investigate the strategies used in talking with parents about diagnosis and longer term outcome.

Tools have been devised, mostly for use in adult health care, to support assessment of patient-clinician communication. These tools include checklists of approaches, behaviours and responses and specifically devised frameworks [12–15]. The Paediatric Consultation Assessment Tool (PCAT) [16] is a rating scale focusing on six aspects of the communication process with children and their parents during consultations. No such tools have been identified which relate specifically to communication between parents of young infants and clinicians. An initial aim of this study was thus to develop a framework to describe the communication between clinicians and parents of preterm infants about brain ultrasound and MRI imaging at term age.

The present study was undertaken as part of a larger research programme on neonatal brain imaging in which a key component was a trial of information-giving to parents following MRI or ultrasound scans at term, of babies born before 33 weeks gestation. The hypothesis of the larger study concerned the effect on parental wellbeing of the more detailed prognostic information provided by MRI. The purpose of this qualitative component was to focus on communication with parents following brain MRI or ultrasound scan. A specifically developed framework was devised to describe the strategies, content and language used in talking to parents.

## Methods

### Context

UK, the ePrime study, a programme of research evaluating the use of MRI to predict neurodevelopmental impairment in preterm infants. The programme of work was approved by Hammersmith, Queen Charlotte's and Chelsea Research Ethics Committee - 09/H0707/98.

### Sample

Parents of preterm infants born before 33 weeks gestation who delivered in 13 NHS trusts in the London area, who had consented to participate in the main study of information-giving following MRI or ultrasound imaging at term age. Most parents or ‘family units’ consented to participate in the audio-recording sub-study (80 % of those participating in the main study, 350 out of 434).

### Procedure and data collection

Parents who had been recruited to ePrime were invited to the diagnostic scanning day at the main study centre. Written informed consent was obtained at the recruitment site a few weeks before the scanning appointment. Prior to the ultrasound and MRI scanning of the baby, parental written consent to the study was affirmed including the audio-recording part of the study. Randomisation to either ultrasound or MRI results sharing took place after both scans had been undertaken. Audio recordings were initiated by one of three clinicians undertaking the discussions during which parents were shown images of the MRI or ultrasound and the findings were described. The scans were carried out immediately prior to the meeting. The results of the scans were therefore only made available to the clinicians after the imaging and just prior to the discussion with parents. Copies of the MRI or ultrasound images were given to parents on the day of the scan and a letter detailing the points made in the discussion was sent to them. A topic guide / script ensured essential information was given in the generally agreed order (Additional file 1: Appendix 1) and images from the scan were used to facilitate the communication process. A total of 60 discussions were recorded, transcribed and analysed. Audio recordings were made of consecutive individual clinician-parent discussions over three specific time periods: during the early, middle and late phases of recruitment and data collection. These time points were chosen to encompass any differences over the course of the study that might occur in the content and style of the discussions. Parents who did not consent to the audio-recording continued to participate in the main study and received their baby’s scan result (MRI or ultrasound) as described above but their discussion with the clinician was not recorded.

### Data analysis

An experienced qualitative researcher (MH) transcribed the audio recordings. The first phase of data collection resulted in the recording of 24 discussions. A preliminary analysis of these was undertaken using NVivio10. Sections of the text were coded in accordance to issues identified in the data. The codes were then organised into themes and sub-themes. New codes, themes and sub-themes were created when the data appeared to capture something new. This process continued until all transcripts had been coded. The themes and sub-themes were then reviewed by MH and MR and combined as appropriate. This analysis was then reviewed by both researchers in the context of more general clinician-patient and clinician-parent interaction studies [12–18], in order to finalise the framework (Additional file 1: Appendix 2).

This specifically devised framework facilitated a further more detailed qualitative analysis also using NVivo10, of 36 recordings (12 from each clinician) taken from across the three periods of recording. Thematic analysis was undertaken [19] supported by the framework. Reading the transcripts, coding and re-reading of these by both researchers in an iterative manner facilitated the identification and sharing of themes and subthemes. The arrangement and grouping of themes and sub-themes was discussed and agreed by both researchers. Saturation with no further themes identified, was reached by the time 36 interviews had been analysed [19].

## Results

Most mothers in the study were in their early 30’s, less than half had previously given birth, most were partnered and half were from Black and Minority Ethnic groups (Table 1). The 36 parental interviews concerned 43 infants: 23 boys and 20 girls, with a mean and median gestation at birth of 30 weeks (range 25 weeks + 2 days to 32 weeks + 6 days). Of the 36 interviews, 19 were with parents whose babies were allocated to MRI and 17 were assigned ultrasound based information. Most parents had singletons (30 out of 36), with 5 sets of twins and one of triplets. Scans took place at a mean of 12 weeks actual age (median, 12 weeks 2 days) and at a mean of 3 weeks corrected age (median 2 weeks, 5 days), with a total 5 out of 43 infants identified as having abnormal scans. Almost all of the discussions (duration 6–48 min, mean 25, median 21 min) took place with mothers present (35), half with fathers (18) and one with a grandmother present. Discussions about MRI results were longer (median: MRI - 16 min, US - 11 min), as were discussions involving multiples (median: multiples - 17 min, singletons - 12 min) and abnormal results (median: abnormal - 26 min, normal - 11 min). Following the discussion, parents were offered a copy of the audio-recording, 28 accepted and these were sent by post.

**Table 1:** Characteristics of mothers whose babies were discussed in the audio recordings study

Maternal characteristics		
Mother’s age (years) *n=36*	Mean	31
	Median	32
	Range	30–51
Previous children *n=34*	No	19
	Yes	15
Partnership status *n=34*	Lives with partner	29
	Does not live with partner	5
Ethnicity *n=34*	White	17
	Black and Minority Ethnic	17
When left education *n=34*	Age16 years or less	4
	Age > 16 years	30
Employment *n=32*	Paid work	6
	Maternity leave	15
	Looking after family	10
	In education/unable to work	3

Within the over-arching theme of clinician strategies four key themes were identified, each of which contained a number of sub-themes. These are listed in Table 2 and are discussed individually with verbatim excerpts given to illustrate.

**Table 2:** Themes relating to the strategies used by clinician during information-giving

*Themes*	*Clinician strategies*
*Framing the information-giving*	Orientating
	Asks what parents know
*What we are looking at*	Uses analogies
	Introduces and explains terminology
*Presenting the numbers and explaining risk*	Paces the information
	Checks understanding
	Invites clarification or questions
*Appreciating the position of parents*	Personalises information
	Emphasises positive
	Recaps
	Answers parents’ questions

### Framing the information-giving

The clinicians started off by describing a plan for the discussion, orienting parents to the objective and framing the discussion in the context of information parents had previously been given about their baby. Whilst the same approach to framing the discussion was taken, the importance of the information to be given was often emphasised when abnormal results were given:

*CL2:* And the way I give the results is that I will first talk about the background at the hospital type of thing-

M: Ok.

CL2: −and then talk about babies born before 33 weeks and then give you the results and what they mean, ok?

***2047***–***2059: 28***^***+6***^***weeks, MRI, normal***

*CL3: …. We do have some results for you today, which I think are important and which I think will be important for the future.... I’m going to tell you some things that you may know already and then I’ll give you the results at the end.****2891: 32***^***+1***^***weeks, MRI, abnormal***

There was active positioning of the information in orienting to the topic. A key element was checking on the information parents had previously received and what they already knew. The clinicians were aware of difficulties that could arise for parents receiving information from different sources and the anxieties this could provoke. The emphasis was on how the babies were now, ‘today’. This contextualising allowed the issue of different and possibly contradictory information, to be handled.

*CL3: …first of all I’d like to find out from you what you know about already about the scans, what you’ve heard from the other hospital and what you think you’re going to hear, so to speak.... It’s also fair to say that some problems don’t show themselves on earlier scans so if there are differences between this scan and what you’ve heard before then that’ll be the sort of reason why.****2106/2131/2144: 28***^***+6***^***weeks, US, normal***

### What we are looking at

In introducing the process of reviewing the scans and sharing the images with parents the clinicians started by using analogies to help them describe the whole brain. In doing this they focused on the shape and the surface before explaining the complex and varied images arising from the scanning process. Analogies commonly used included ‘walnuts’, ‘carpets’ and ‘railway junctions’. There were pauses for parents to respond and the emphasis was on what ‘we’ and ‘you’ can see, which parents acknowledged in a fairly minimal way:

*CL2: Ok. So when we, when we look at the brain through the scans, we look at the surface of the brain-*

*F: Yes.*

*CL2: −which is called the cortex, which is folded like a walnut.****1435: 29***^***+6***^***weeks, US, normal***

*CL1: As you can see the surface is not smooth, can you see, it’s folded?*

*M: Yes.*

*CL1: It’s like a carpet. Somebody has walked on a carpet and it’s got folded. So it’s folded up and down. That’s how a normal brain looks like.****6705: 25***^***+4***^***weeks, MRI, normal***

*CL1: And those are kind of like junctions, like, imagine Clapham Junction, it’s kind of taking signals from the surface of the brain and then deciding where else the signals should go to.****7595: 29***^***+2***^***weeks, US, normal***

The clinicians also acknowledged the difficulties parents might have in seeing any detail in the images shown, implicitly contrasting parents’ position with their own based on medical knowledge and experience. They again often used analogies to help reassure parents about what they were seeing:

*CL3: And I’m going to show you an ultrasound scan which I’m sure you’ve seen before…. so that we can know what we’re talking about. And I don’t know if you’ve seen these before?*

*M: No.*

*CL3: Ok, well in that case, I know it looks like a sort of fuzzy snowstorm.*

*M: Yes.*

*CL3: But there is quite a lot of information in here.****1636: 30***^***+0***^***weeks, US, normal***

The clinicians then introduced and explained medical terminology to describe the developing brain as they talked. They referred to structures, sections and slices in describing what parents were being shown. Specific structures are named, with some references to function. Much of this seemed to be aimed at helping parents to get used to looking at the scans and acquiring a vocabulary. This also prepared them for what they would see, in order to understand what may have happened and the prognostic information the clinician was planning to give. The same approach was taken irrespective of whether normal or abnormal results were given:

*CL2: So we’re starting from the base of the neck now, ok? So this part, this part here is called the cerebellum.*

*M: The cerebellum?*

*CL2: Yes and we think it’s important in terms of movement, you know, in walking and things and also in terms of memory. So the way that it looks, it’s like we want it to be like, ok? So this is fine, ok?*

*M: Ok.*

*CL2: Then as we start to look here, if you see this black thing here.*

*M: Yes.*

*CL2: That’s just a vessel. So it’s like a vein, where the blood flows. So everywhere in our body, blood flows.*

*M: Ok.*

*CL2: So because this is an MRI, we’re able to see it, just like you can see, there.*

*M: Ok.*

*CL2: So that’s just a vein, a vein where the blood goes.*

*F: Yes.*

*CL2: So this is not because of something wrong with the cerebellum, we’re just seeing it-*

*M: Oh.****4986: 32***^***+6***^***weeks, MRI, abnormal***

*CL1: …. we’re going to talk about the brain and then I’m going to show you the ultrasound pictures. So before I do that I’m going to talk a little bit about the brain structures so I can point it out on the ultrasound when I show it you. So I’m sure you know that the brain, it has two sides, right and left and there are actually fibres connecting the two sides called the corpus callosum. There’s no need to know that. But there are fibres. Obviously they intercommunicate….****9664: 32***^***+1***^***weeks, US, normal***

Clinicians explained that images can look different depending on the section of the image being viewed. Nevertheless, using these different but related images when talking to parents was not easy:

*CL2: So, what I’m going to do now, is just to take you through the pictures I’ve taken and answer those other questions that you had about the scan that they did. These pictures, I’ve taken them in the same way as the picture I’ve just given you, ok? But I’m starting from the forehead and am working my way to the back and then I’ll tell you when we’ve turned and are looking at things from a different angle….****4327: 27***^***+1***^***weeks, US, normal***

The difficulties clinicians experienced describing the images they were sharing with parents and explaining the principles were evident in the mixture of lay and medical terminology:

*CL2: Now the white matter is the tissue that is immediately vulnerable in preterm babies to having problems. So that’s where we usually see problems, ok? And then the brain itself also has natural cavities called ventricles, into which sometimes there might be bleeding which we can see on the scans. And when there is bleeding, sometimes the ventricles themselves might get a bit bigger.****2173: 30***^***+0***^***weeks, US, abnormal***

In introducing medical terminology to parents clinicians also used analogies to describe the key features of the brain structures they were going to refer to when looking at the different cross-sectional images. At the same time as using such terminology, they also used language that would have been more familiar, identifying discernible shapes such as a ‘tear drop’, ‘commas’ or ‘ticks’, a ‘moustache’ or a ‘blade’:

*CL2: And then the natural cavities which are called ventricles, at the front they look comma shaped like that, and at the back they’re teardrop like.****7522: 32***^***+5***^***weeks, MRI, normal***

*CL1: …. if you can imagine, it looks like a flat blade…. From the front, it actually goes back and then somewhere in the middle it joins together, and then it comes down to the third ventricle and then the forth ventricle. We actually have four ventricles. These are the right and left and then third, that is when they join together and the fourth is right in between the brain stem.****9569***–***9576: 30***^***+5***^***weeks, MRI, normal***

In viewing the scans the clinicians also recognised the issues associated with identifying structures, referring to the need for parents to ‘imagine’ and ‘believe’ in what is being identified, trusting what the clinician is describing:

*CL1: ….these two areas, you have to trust me, to believe me, there, because that’s what ultrasounds are like, that grey area there is the basal ganglia and thalami. Trust me, it’s not very clear to you, I’m sure it isn’t, but this is where it normally is. And on top of it, you know this little, like two ticks, two black ticks, this one?*

*M: Yes.*

*CL1: That is the ventricle, the fluid filled space.****7646****–****7633: 29***^***+1***^***weeks, US, normal***

The emphasis from the clinicians on the ventricles as ‘natural cavities’, gives the clear message that they should be there, while at the same time recognising that depending on the section, the ventricles can look different in terms of size and shape. Underpinning this is the knowledge that these structures can be damaged in preterm infants in a way that is particularly important in functioning brains. Clinicians also used analogies to explain the function of different structures and what has been observed:

*CL2: Actually, where the ventricles are, there’s a water that cleans our brain so the ventricles are like the drainage system of the brain.*

*F: Yes.*

*CL2: It’s pretty much like you have at home. So if for instance, in your kitchen when you wash our dishes, you leave food, it’s going to block there. So that’s what the bleed will do. So if it’s a big one, it will block the outgoing water that’s cleaning your brain. So there will be a build up behind, so just like in your kitchen, if you leave the food as you’re cleaning the dishes there’ll be water left behind until you unclog it.*

***6125: 31***^***+5***^***weeks, US, normal***

*CL1: … there’s another big clump of cells in the centre of the brain called the basal ganglia and thalami which I will try and show you on the image afterwards. So, these are very important site in the brain because they’re kind of like a junction determining where signals coming in should go, you know, like that train junction?*

***7501: 25***^***+2***^***weeks, US, normal***

Both parents and clinicians referred to how the brain looked and to specific features. In explaining small differences and normal variation to parents in relation to symmetry, clinicians made efforts to normalise what was observed:

*CL3: … there’s nothing we know about that having that slight enlargement that says this is bad…. it’s a bit like looking at somebody’s face, you know, my eyes are a little bit lopsided, but I can still see perfectly well through them. There are differences between people.****1622: 28***^***+2***^***weeks, MRI, normal***

*F: … about the, the dysymetry in each of their brains, of the ventricles… I mean, when the initial scan, it didn’t look like it was a normal thing, so is it pretty normal in terms of statistics?*

*CL2: It didn’t look normal?*

*F: No, it’s not completely symmetrical in their brains and I don’t know if it’s pretty standard …*

*CL2: It is. It is a usual thing to see. Just like all of us are never symmetrical. Your heads are never the same size. Nothing in us is completely symmetrical …. So it’s a completely acceptable finding.****2047–2059: 28***^***+6***^***weeks, MRI, normal***

This occurred with parents whose babies were described as having brains that looked ‘normal’ and those about whom there was concern and a poorer prognosis.

### Presenting the numbers and explaining risk

In talking to parents about possible future outcomes for their children clinicians have the inherently difficult task of presenting ‘risk’ and probability data effectively. Strategies used by clinicians to overcome these challenges included pacing the information, checking understanding and inviting questions. Addressing the question of prognosis and predicting outcome, they set the scene overall, provided some figures and reframed in relation to the individual scan findings:

*CL1: .... So, being born preterm puts you at some risk for some problems and one, which is talked about a lot, which is the problem called cerebral palsy, which is a motor problem. I’m not saying he’s going to get it.*

*M: No, no, no.*

*CL1: I’m just explaining the risk of the whole picture of preterm. It depends on how preterm you are. Obviously, the more preterm you are, the higher the risk. So if you’re born below 29 weeks, then your risk can be up to about 14 %. But if you’re between 29 and 33 weeks then it’s about 6 %, the risk. But overall, anybody born preterm below 33 weeks, it’s got a 9 % number that everybody quotes. Ok? So, but with the normal scan today, it just means that the risk is brought down tremendously to only about 2 %.***6756:*27***^***+4***^***weeks, MRI, normal***

Information-giving and presentation of the risks of different outcomes such as cerebral palsy took place with all parents, using both numbers and percentages. Recapping and referring back to earlier scans and previous information-giving by clinicians in other settings were common strategies. Not all parents had an understanding of the potential consequences of preterm birth. The clinicians checked on parent’s awareness and knowledge of possible outcomes and then elaborated. This was done by providing further information about the risks and the pattern of development for this group generally and then for their baby using what was found in the scans:

*M: Like what are you saying then, that she’s got cerebral palsy?*

*CL2: … just by being born before 33 weeks they have a 9 % chance of having cerebral palsy… and she was born at 29 weeks, so the risk goes down to about 6 %. That’s just by being born. So it has nothing to do with your scans or anything else…… now that we have scan we can update that information and say, based on the scan result that we’ve got, and the scan result has this combination of changes. So with this combination of changes her risk of having cerebral palsy has gone up to about a third. So that doesn’t mean she is going to have [CP]. It means there is a chance that she might have.*

*M: When will that manifest itself?*

*CL2: By about two years corrected age is around the time that we assess for that properly. Some people can say around one year corrected age they can start assessing for it. But it’s about two years corrected age that we can be categorical that she’s got it or not.*

*M: So when you say filling up (the ventricles), what, does that mean that the risk has gone up for her having cerebral palsy.*

*CL2: The risk of cerebral palsy is as a result of the combination of the things, it’s not the one thing.*

***1365: 29***^***+0***^***weeks, MRI, abnormal***

*M: So you can’t say at this stage that he definitely won’t have any issues. You can just lower the risk, right?*

*CL3: Yes. So the risk, his risk was almost 10 %, so one chance in ten.*

*M: Yes.*

*CL3: With a normal scan it reduces to as low as 2 % or two chances in a hundred or perhaps as high as 6 % depending on how accurate we can be. Certainly less than it would be if the scan was not normal.*

***5452: 31***^***+3***^***weeks, MRI, normal***

In pacing and structuring the information clinicians used reference points in conveying information about risk or the ‘chance’ of future problems. Comparisons were made across different gestational age groups and term babies in trying to present and make sense of the boundaries of ‘normality’ or ‘normal’ in relation to the imaging and the probability of a good or poor outcome:

*CL3: ..... we can tell you quite precisely that there’s no more chance than a 6 % chance of having problems with that, having problems with moving or walking and things like that.......*

*M: That’s great. How does that then, 6 % compare to a full term baby?*

*CL3: Ok, so we say, full term babies, it’s about 2 %.*

*M: Ok. So it’s a bit increased*

*CL3: …. So, so it’s not quite 2 % cerebral palsy, but 2 % for any problems.****1784: 30***^***+4***^***weeks, US, normal***

*M: When you say like, within the normal limits for what you’d expect preterm-*

*CL3: Yes.*

*M: Is that different like, does that differ a lot to term?*

*CL3: It can do, but the…. preterm babies who have slightly large cavities ….have the same outcomes as term babies who don’t have large cavities, as far as we can tell****1622: 28***^***+2***^***weeks, MRI normal***

The complexity of the kind of prognostic information and neurological features on which this relies is reflected in the clarification required, language used and the reminder that there is no absolute certainty about outcome:

*CL2: …. we could never be 100 % certain because, again we can’t be certain there’s nothing definite on there that says if you have this, you will never have that.*

*M: So that’s more like our presentation.*

*CL2: The risk becomes diminished if, you know, things look a certain way. But it’s all about observing certain kind of things and if those kind of things are picked up, then you know that there might be a problem ….*

***2047***–***2059: 28***^***+6***^***weeks, MRI, normal***

The incidence of future problems in term babies was a key comparison reference point for clinicians and parents:

*M: Do all term babies, I mean what’s the risk percentage for term babies to have cerebral palsy?*

*CL1: It’s usually about 1 %.*

*M: 1 %.*

*CL1: Yes.*

*M: So X ((baby)) is not far from that at 2 %.*

*CL1: Yes.*

*F: Yes, it’s still low isn’t it, 2 %, I mean it’s not too-.....*

*M: So the fact that X ((baby)) has a normal scan, we hopefully won’t have anything to worry about?*

*CL1: No, that’s right, the risks are extremely low right down to near to normality*. ***7519: 26***^***+2***^***weeks, MRI, normal***

The clinician in the following excerpt references normality where possible, to present the likelihood of an adverse outcome and to check understanding. However, parents may have difficulty in reconciling themselves to this, particularly when their baby’s condition seems to have improved:

*CL3: Yes, 32 weeks [gestation], then the chances of having cerebral palsy when you grow up is around 4 %, so about four babies in every 100 born at that age will have cerebral palsy when they grow up. Do you know what cerebral palsy is?*

*M: What?*

*CL3: Ok, let me, let me explain. Some children, when they grow up have some problems with movement, moving arms, …moving legs, and that’s usually caused by what we call cerebral palsy......*

*CL3: The scanner has picked up a bit of brain that has died. It’s quite small piece of brain, but there is a little bit of brain there which has died as a result of the sickness that your baby had.*

*M: He’s not sick now.*

*CL3: Well, he’s not sick now, he’s well now but before, he was very sick*

*M: Ok.*

*CL3: So as a result of being sick and premature, this little bit of brain has died. And as we come further down, it’s back to normal again…. Now, this does have consequences for your baby. This will affect probably how-*

*M: What?*

*CL3: This will probably affect how the baby grows up .... although the baby will grow up being able to see, I think, very well, it may be different from the way that other people see and it’s very important that your paediatrician knows about this so that they can help you look after the baby in the years to come. ....*

**2891: 32**^**+1**^**weeks, MRI, abnormal**

Most of the time the clinicians are utilising the probabilities based on population based research evidence to inform parents about the possibility of a poor outcome, while recognising that predicting the ‘chance’ or probability for the individual at this stage of life is fraught with difficulty.

### Appreciating the position of parents

The complexity and multifaceted nature of the interaction with parents in giving technical, descriptive and prognostic information is reflected in this theme. Strategies included personalising the information, emphasising when positive information was being given, recapping information and answering the parents’ questions. Talking to parents as individuals and personalising the information about their baby was an important starting strategy in engaging them in what could be a difficult conversation, particularly when an abnormal result was to be given or when there were twins or triplets:

*CL3: So I want to show you your babies’ scans…. now, let’s get them right, shall we. Shall we put them in order of where they are in the room?....So let’s start with X ((baby 2)). Actually his scans have been easier to see than the one we looked at before…, there’s a fissure down the middle, there’s the cavities. They’re a little bit easier to see than the one we showed you before and that looks absolutely normal…. you can see how these things vary because actually X’s ((baby 3)) is slightly different and the cavities are not quite so prominent…*

*M: Yes.*

*CL3: …they’re even smaller still. That’s not a significant difference, that’s just normal variation, there’s plenty of that and you can see that actually the structure in general looks a bit different. That’s partly because this is taken at very slightly different angle from that one…. it’s always a little bit different. But those are three very nice, normal scans.****2106-2131-2144: 28***^***+6***^***weeks, US, normal***

The verbal descriptions and explanations that clinicians gave, followed by checks and reiterations reflect the communication issues and dilemmas inherent in expert-lay interactions. When positive news was given the clinicians made overarching statements about ‘very good scans’, a ‘nice picture’, ‘a beautiful scan’ and a ‘nice result’ and that the scan was ‘absolutely normal’. In this context they described themselves as ‘delighted’, ‘pleased’ or ‘happy’:

*F: So the size of his head is what you’d expect it to be?*

*CL2: Yes and that’s all that really matters for preterm babies. So long as they are following the centile charts, then we’re happy.****4316: 30***^***+4***^***weeks, MRI, normal***

*CL3: Ok, well that’s great.... These are the areas that we expect to see problems in some babies and …and that also looks well within normal limits for a child who’s been preterm and got to this stage. There’s nothing on there either that would relate to bad, bad outcomes. So we’re very happy to give you those results....there are also some cavities with fluid in. They should be a certain size, preterm babies often have them bigger than babies born at term. Your little one, pretty, pretty like a term baby, one little bit of it’s a bit bigger than it would be, but then again nothing, nothing that we would say would predict the future as being bad. So, yes, we’re very happy with that and that’s really what we want to tell you.****1622: 28***^***+2***^***weeks, MRI, normal***

A different approach was used in discussions where clinicians had to give more concerning news to parents. The language changed, there was more repetition and recapping, clinicians tried to assess what parents already knew and a sense of putting off the certainty was evident. Reference was also made to confirmation of diagnosis at the follow-up developmental assessment two years on:

*CL2: …there are crops of them [cysts] at the back, compared to at the front.*

*F: So the bits of the brain that’s missing, when you compare the right side to the left side, even if you look around the edges of the skull-*

*CL2: Yes, because, because the brain hasn’t been able now to grow properly….*

*M: …. so you can’t say that’s she’s got cerebral palsy but-*

*F: No-*

*M: −but there’s a chance of her getting that.*

*F: −it’s a percentage chance-*

*M: Or is it that-*

*CL2: So you can’t see cerebral palsy now.*

*M: Right.*

*CL2: You see cerebral palsy as the person develops.*

*M: Develops.*

*CL2: We that think that she’s got a pretty good chance of having cerebral palsy.*

*M: Yes.*

*CL2: But we can confirm that when she comes [for follow-up].****3873: 31***^***+2***^***weeks, MRI abnormal***

Some parents were concerned about features they could see or that had been identified previously. Clinicians made efforts to answer their questions and to emphasise the positive while acknowledging what could still be seen:

*M: And the cyst, you don’t see?*

*CL3: We don’t see a cyst in the brain. We see a little cyst on the surface of the brain, but not in the brain itself and all in all, this says a very low chance of cerebral palsy.*

*M: But I can see [with] my eyes, before, the other hospital-*

*CL3: You could see it.*

*M: −the two small, but this is finished now?*

*CL3: We can’t see them. We can see what might be the result, the end result of that.*

*M: But when we go home, it’s smaller, it’s changing.*

*CL3: Yes. We look at this and we have a set of rules that tell us how to predict the future from this scan.*

*M: Ok.****5021: 29***^***+4***^***weeks, US, normal***

When clinicians raised the issue of the risk of cerebral palsy, they often described the kind of problem that could develop and at the same time framed the scan findings positively. Tension between these positions was evident:

*CL3: Cerebral palsy is a wide and varied thing…. a movement problem.…. The arms and legs go stiff and they don’t move properly. It can be very severe things like that or it can be very mild things, just like a clumsy hand or just a stiffness in walking. But we would predict that she wouldn’t have any of those......So we would say she’s only got between 2 and 6 % of even the mild-*

*M: The mildest of it.****5175: 27***^***+6***^***weeks, MRI, normal***

## Discussion

The discussions and recordings provided a unique opportunity to directly evidence the strategies employed by clinicians in giving prognostic information to a diverse group of parents. The literature identifies some of the challenges and difficulties encountered by clinicians and parents during the provision of information, particularly when this is complex and has far-reaching significance for families [7–9]. Whilst literature regarding clinician-patient and clinician-parent communication was used to develop the initial framework for this study [12–18] no published studies have focused specifically on recorded discussions between clinicians and parents about neonatal brain imaging.

The aim of the discussions with parents was to give results of the scans, to place these in the context of information they had received previously and to present what is known about the associated risks of preterm birth in relation to the findings for their baby [20, 21]. In recording the discussions the objective was to develop the analysis framework. This was used to explore the language used, how information and explanations were given and to describe the clinician strategies used in informing parents about diagnosis and the possible longer term outcomes for their children. We were particularly interested in the way the imaging information was handled, the use of technical terminology and the way that risk was presented.

The themes identified were: ‘Framing the information-giving’ which sets the scene, and the baseline; ‘What we are looking at’, the big issue of terminology which skilled and knowledgeable health professionals have to deal with; ‘Presenting the numbers and explaining risk’ which embodies real and subjective probability issues and ‘Appreciating the position of parents’ which reflects ways in which clinicians responded to parents needs and understanding as individuals.

For clinicians there are difficulties inherent in explaining structures in three dimensions, identifying specific features and integrating this with information describing processes and change over time. Throughout the discussions there was, of necessity, iteration of the information presented in different ways and of key points. Like most parents of babies requiring neonatal care [1, 2, 7, 8] these parents were seeking certainty and reassurance. The clinicians found it hard not to be reassuring, while at the same time trying to give messages about possible outcomes, particularly for those infants with an abnormal scan. For clinicians, familiar with numerical data used as an evidence-base in decision-making, the probabilities they presented reflected their home ground. However, the proportions and probability information were difficult to convey effectively. This suggests that consideration of other modes of communication, particularly visual graphical representation as a way of facilitating discussion and understanding could be worthwhile [22].

There are commonalties with other information-giving situations in neonatal care and paediatrics more broadly in which the aim is also to achieve a shared meaning and understanding [16, 23]. While the study provides a broad description of the content of clinicians' information-giving process, it is difficult to draw strong conclusions about how information should be shared and to provide clear guidance. We would hope that clinicians reading this paper reflect on how they themselves share information with parents. It may be that those whose role involves giving complex information to parents could better plan what they will communicate in advance of meeting individual parents, particularly where the findings are of likely to be of concern. They could also consider shared interviews with another member of the team in addition to utilising other materials, particularly in relation to giving information about risk [22]. The potentially negative implications of this type of intervention [8] also need to be considered in terms of any possible effects on the short term and longer term wellbeing of parents. It is anticipated that the other quantitative components of the ePrime programme will contribute to the evidence base in this regard.

Further analysis is planned of the way in which longer term outcomes are explained and understood. Interviews undertaken later in the study will explore the impact of early information-giving on parents one and two years on from the prognostic discussions described in this paper. Thus quantitative and qualitative data collected at around one and two years after these discussions will further explore the impact of the early prognostic information-giving experience and reflections on these from the parents’ perspective.

### Strengths and limitations

Recordings are rarely made of clinician-parent interviews. The openness and willingness of both parents and clinicians to participate is a strength of the study, as was the unbiased way in which the specific recordings were made. Data collection took place in the context of a research trial using a topic guide and it is acknowledged that this might not reflect what occurs in clinical practice routinely. However, we would argue that many aspects of giving prognostic information to parents of young infants would be similar. Whilst there were some minor differences in approach between the clinicians, it was not considered meaningful to undertake qualitative analysis of these.

Video-recording would have allowed us to appreciate the role of non-verbal interaction and non-verbal cues and how the clinicians used gestures in explain the scans shown to parents. However, this could be considered more invasive and likely to affect parent and clinician willingness to participate. In this analysis the focus was on the strategies used in giving information to parents about the findings from ultrasound and MRI scans and expert examination of these. The study data presented, the framework used and the associated strategies described may enable clinicians to be more conscious of the language and constructs they use in discussions with parents. A more detailed analytical focus on the interaction and communication interface between parents and clinicians could facilitate further understanding of the dynamics and processes involved. The framework developed could be used in other health related situations involving parents. Further quantitative research could explore different methods of information provision in this kind of context.

## Conclusion

Clinicians used a range of strategies in communicating key information to a diverse group of parents. Clinicians recognised parents as individuals who brought different experiences and levels of knowledge and understanding to the discussion. The themes identified reflect the practical difficulties of giving prognostic information to parents based on brain imaging. The insights gained may encourage health professionals working with families to reflect on and possibly modify their own practice.

## Additional file

Additional file 1: Appendix 1. Topic guide used to facilitate the provision of essential information. **Appendix 2:** Framework developed for analysis of audio-recordings based on first 24 recordings and list of sources. (DOCX 25 kb)
