# Supplementary material for: Explanations and information-giving: clinician strategies used in talking to parents of preterm infants
Source: BMC Pediatr. 2016 Feb 11;16:25. doi: 10.1186/s12887-016-0561-6 (PMC4750359; doi:10.1186/s12887-016-0561-6)
Supplement: Additional file 1: Appendix 1. — Topic guide used to facilitate the provision of essential information. Appendix 2: Framework developed for analysis of audio-recordings based on first 24 recordings and list of sources. (DOCX 25 kb) [file 12887_2016_561_MOESM1_ESM.docx]

**Appendix 1** Topic guide used to facilitate the provision of essential information

| - Randomisation and how the results will be given - What parents have previously been told about scan results and the baby’s prognosis - An overview of the MRI or ultrasound result - More detailed information about the scan using the images to explain the findings - General long-term risks of problems for babies born preterm with specific reference to cerebral palsy and learning difficulties - Prognosis for the baby based on the scan result, with reference to risk of cerebral palsy and learning difficulties |
| --- |

**Appendix 2:** Framework developed for analysis of audio-recordings based on first 24 recordings and sources (1-9) as listed below

| Sub-themes | | Key Theme |
| --- | --- | --- |
| **Facilitating the communication** | | The communication interface |
| Sourced from the preliminary analysis: | Sourced from the literature: |  |
| Clinician demonstrates empathy  Clinician makes supportive responses  Clinician responds to parents’ cues  Clinician explores parents’ issues and concerns  Clinician acknowledges parents’ worries and concerns  Getting sex or name of the baby correct  Allowing parents to speak without interruption | Clinician demonstrates concern (2, 3, 7)  Clinician is reassuring (2, 3, 7, 9)  Patient (parent)-centredness (2, 3, 5, 7, 8)  Clinician asks parents their opinion (2, 3, 7)  Clinician compliments parents (2, 3, 7)  Clinician uses an open response (5, 6)  Clinician asks parents about their expectations or feelings (5) |  |
| **Hindering the communication** | |  |
| Sourced from the preliminary analysis: | Sourced from the literature: |  |
| Giving rushed, muddled or ambiguous information  Uses complex terminology without clarification  Clinician did not invite questions  Parents’ questions not answered  Clinician did not check understanding  Getting sex or name of baby wrong  Overlapping speech  Missing out key elements of the interview | Negative talk (2, 3)  Disagreement between clinician and parents (2, 3)  Clinician does not respond to parent’s questions or cues (5)  Uses complex terminology without clarification (6)  Clinician uses a closed response to parent’s questions (5)  Clinician interrupts parent (6)  Lengthy information with limited opportunity for parental response (9) |  |
| **Clinician strategies to help parents understand**  Sourced from the preliminary analysis: | Sourced from the literature: | Reaching an understanding: clinician strategies |
| Personalising information  Follows the topic guide  Sign-posting and orientating information  Using diagrams, pictures and analogies  Providing supplementary information  Inviting questions  Repeats information  Checks understanding | Engages parents (1, 9)  Asks parent what they already know (4)  Sign-posting and orientating information (1, 2, 3)  Adapts information to meet the needs of parents (1, 4)  Inviting questions (2, 3, 9)  Uses open, appropriately worded questions (1, 9)  Answers parents’ questions (4)  Summarises and paraphrases information (1)  Checks understanding (2, 3) |  |
| **Prospects for the baby:** | | Looking to the future |
| Sourced from the preliminary analysis: | Sourced from the literature: |  |
| Information giving about diagnosis and prognosis  Clinician explains what information means for the baby / family | Health education (2, 3, 7)  Biomedical information (2, 3, 7) |  |

Literature sources used in developing framework for analysis of audio-recordings

1. Howells RJ, Davies HA, Silverman JD, Archer JC, Mellon AF (2010) Assessment of doctors’ consultation skills in the paediatric setting: the Paediatric Consultation Assessment Tool Archives of Disease in Childhood 95 323 - 329
2. McCarthy DM, Buckley BA, Engel KG, Forth VE, Adams JG, Cameron KA. (2013) Understanding patient-provider conversations: What are we talking about?

Academic Emergency Medicine 20 441-448

1. Roter D, Larson S (2001) The relationship between Residents’ and Attending Physicians’ communication during primary care visits: An illustrative use of the Roter Interaction Analysis System Health Communication 13 1 33-48
2. Lehmann F, Côté L, Bourque A, Fontaine D (1990) Physician-Patient Interaction: A reliable and valid check-list of quality Canadian Family Physician 36 1711-1716
3. Henbest RJ, Stewart M (1989) Patient-Centredness in the Consultation 1: A method for measurement Family Practice 6 4 249 - 253
4. Roter D, Frankel, R (1992) Quantitative and qualitative approaches to the evaluation of the medical dialogue Social Science and Medicine 34 10 1097 - 1103
5. Roter D, Larson S (2002) The Roter interaction analysis system (RIAS): utility and flexibility for analysis of medical interactions Patient Education and Counseling 46 243 - 251
6. Henbest RJ, Stewart M (1990) Patient-Centredness in the Consultation 2: Does it really make a difference? Family Practice 7 1 28 - 33
7. Shilling, V. Williamson, P.R. Hickey, H. Sowden, E. Beresford, M.W. Smyth, R.L. Young, B. (2011) Communication about children’s clinical trials as observed and experienced: Qualitative study of parents and practitioners PLoS One 6 7 e21604
